# Emerging Challenges and Advances in Porcine Circovirus: A Decade in Review

**DOI:** 10.1155/tbed/4921135

**Published:** 2026-01-19

**Authors:** Jiawei Zheng, Guoqing Zhang, Peiheng Li, Linzhu Ren

**Affiliations:** ^1^ College of Animal Sciences, Key Laboratory for Zoonoses Research, Ministry of Education, Jilin University, Changchun, 130062, Jilin, China, jlu.edu.cn; ^2^ State Key Laboratory for Diagnosis and Treatment of Severe Zoonotic Infectious Diseases, Jilin University, Changchun, 130062, Jilin, China, jlu.edu.cn

**Keywords:** capsid protein, immunosuppression, pathogenesis, porcine circovirus, vaccine

## Abstract

Over the past decade, porcine circoviruses (PCVs) have continued to pose a significant threat to global swine health, and pivotal discoveries have significantly reshaped our understanding of their biology and control. Extensive genomic surveillance has expanded porcine circovirus 2 (PCV2) genotyping from four to at least eight lineages, with PCV2d now globally dominant under vaccine‐driven selection pressure. Since 2016, three novel species, PCV3, PCV4, and PCV5, have been identified, linked to reproductive failure, myocarditis, multisystemic inflammation, and potential neuroinvasion; however, their pathogenic potential remains under active investigation. Recent studies have revealed that PCVs evade host defenses by targeting the cyclic GMP‐AMP synthase (cGAS)–stimulator of interferon genes (STING)–type I interferon (IFN‐I) pathway and modulating regulated cell death pathways, thereby fostering viral persistence and immune dysregulation. PCV–induced immunosuppression not only exacerbates bacterial and viral coinfections but also impairs vaccine efficacy, leading to complex clinical outcomes. Advances in structural virology have clarified the roles of the Cap protein, identifying key antigenic loops and posttranslational modifications that influence immunogenicity and vaccine escape. This knowledge has accelerated the development of novel diagnostic assays and next‐generation vaccines. Furthermore, vaccine innovation has progressed beyond traditional inactivated formulations to recombinant subunit, virus‐like particle, and DNA platforms, some of which incorporate modular or multivalent designs to address genotype diversity and coinfection scenarios. Despite these advances, challenges persist, including the continuous emergence of immune‐escape variants, inconsistent vaccine performance under field conditions, and an incomplete understanding of the pathogenicity of PCV3 to PCV5. Therefore, multidisciplinary strategies integrating molecular epidemiology, structural vaccinology, and advanced biotechnologies will be critical to closing current knowledge gaps and ensuring sustainable PCV control.

## 1. Introduction

Porcine circoviruses (PCVs) have emerged as major pathogens in the global pig production over recent decades, causing PCV–associated diseases [1]. PCV–associated diseases manifest as multiple clinical entities, including PCV2‐systemic disease (PCV2‐SD), porcine dermatitis and nephropathy syndrome (PDNS), reproductive disorders, and respiratory illness [1]. The overall burden on health and productivity is considerable: pigs grow more slowly, feed conversion worsens, and mortality increases, together driving substantial economic losses [2]. These losses arise not only from death and reduced performance but also from heightened spending on veterinary services, diagnostic testing, and vaccination [3]. In some regions, annual economic losses from PCV–associated diseases have been estimated at hundreds of millions of dollars [3].

Over the past decade, intensive research has advanced our understanding of PCVs and yielded more effective control strategies [1, 4]. New findings have shed light on viral genetic diversity, pathogenic mechanisms, immune evasion tactics, and the development of improved diagnostic tools and vaccines [5, 6]. This review summarizes key advances and emerging challenges in PCV research over the past decade.

## 2. PCV Pathogenicity and Prevalence

### 2.1. Classification and Characteristics of Porcine Circovirus

#### 2.1.1. Current Taxonomy and Genomic Features

PCVs are tiny, nonenveloped viruses with circular single‐stranded DNA genomes, typically 17–25 nm in diameter, ranking among the smallest known animal viruses [1, 7–9]. Five recognized species, PCV1 through PCV5, have been identified. PCV1 was initially discovered as a nonpathogenic contaminant in the porcine kidney cell line PK‐15. PCV2, the etiological agent of PCV–associated diseases, exhibits genotypic diversity. Whereas only four genotypes (PCV2a, PCV2b, PCV2c, and PCV2d) were recognized a decade ago [7–9], eight genotypes (PCV2a–PCV2h) are now formally defined based on phylogenetic and genetic distance analyses of the open reading frame 2 (ORF2) gene [10], with a potential ninth genotype (PCV2i) also proposed [11]. These genotypes exhibit distinct global distribution patterns and well‐documented shifts over time. Evidence suggests that some lineages, such as PCV2d, may exhibit greater replication fitness and partial immune escape than earlier vaccine‐derived PCV2a strains. However, marked differences in intrinsic pathogenicity across all genotypes have not been consistently demonstrated. Notably, PCV2b overtook PCV2a around 2005 as the dominant lineage, and PCV2d has since become globally prevalent, likely driven by immune pressure from widespread vaccination [12].

In recent years, metagenomic sequencing and molecular surveillance have led to the identification of three novel PCV species, PCV3, PCV4, and PCV5 [13–15], further expanding the known diversity of the PCV family; however, evidence regarding its prevalence, host range, and pathogenic relevance remains extremely limited. PCV3 was first identified in 2016 in the US and has been linked to reproductive disorders, myocarditis, and systemic inflammation in pigs [13]. Phylogenetically distinct from PCV2, it shares only ~48% genome‐wide nucleotide identity with PCV2 [13]. Like PCV2, its genome encodes three major ORFs but with substantial sequence divergence.

PCV4 was subsequently detected in 2019 in China in pigs showing respiratory and gastrointestinal symptoms [14]. Similar to PCV3, it exhibits low nucleotide identity with existing PCVs (~43% with PCV3) and forms an independent clade [16]. These discoveries were enabled by next‐generation sequencing (NGS), which uncovered previously undetectable circoviruses in pig populations. However, their pathogenic potential and epidemiological impact remain under investigation. Since its discovery, PCV4 has been reported in South Korea, the US, Thailand, and parts of Europe, but its transmission dynamics and prevalence remain poorly understood [17–20].

More recently, a novel circular single‐stranded DNA virus, tentatively designated PCV5, has been identified in pigs with respiratory, diarrheal, and reproductive disorders through metagenomic surveillance [15]. PCV5 possesses a larger circular genome (~2.9 kb) encoding Rep and Cap proteins with very low amino acid identity to those of PCV1–4. Phylogenetic analyses based on Rep place PCV5 outside the family Circoviridae, clustering instead with fur seal feces‐associated circular DNA virus, suggesting that PCV5 represents a distinct lineage within the broader group of CRESS DNA viruses. Notably, PCV5 has been detected at relatively high molecular and serological prevalence in pigs from southern China, and infectious virus and virus‐like particles (VLPs) have been successfully generated in vitro. However, evidence regarding its transmission dynamics, host range, and causal role in PCV–associated diseases remains limited, and its taxonomic status and pathogenic significance require further independent validation.

**Figure 1:**
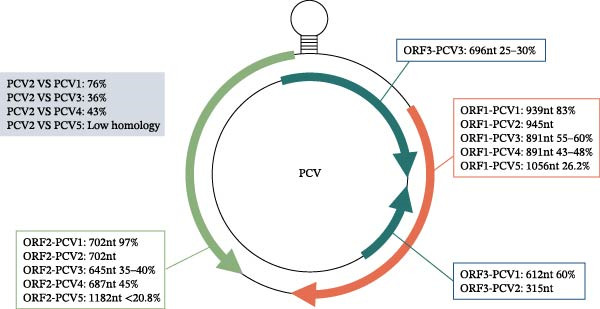
Comparative genomic organization and nucleotide identities of PCV1–4. Schematic representation of the circular PCV genome highlighting the three major ORFs. The lengths and nucleotide identities of ORF1, ORF2 (Cap), and ORF3 are shown for PCV1–4, with percentage identities relative to PCV2. The blue box denotes whole‐genome nucleotide identities between PCV2 and other PCVs, illustrating the close relationship with PCV1 and the more distant divergence of PCV3 and PCV4. The figure emphasizes the high conservation of ORF2 between PCV1 and PCV2 and the substantial sequence divergence of PCV3 and PCV4 across all ORFs.

Notably, while PCV3 and PCV4 share the three major ORFs of PCV2, the functional conservation of these ORFs, particularly with respect to replication efficiency and immune modulation, remains unclear. For a visual summary of genomic characteristics and sequence similarities/divergences among PCV1‐5, see Figure 1, which presents overall genome identities and ORF–specific nucleotide comparisons.

#### 2.1.2. Evolutionary Origins and Phylogenetic Analysis

PCV2, the first pathogenic PCV identified, was retrospectively detected in tissues dating back to the 1960s, although its evolutionary origin is considerably older [21]. Phylogenetic analyses indicate that PCV2d emerged in Southeast Asia between 2008 and 2011, then spread to China, where it became the dominant circulating strain [22]. The virus exhibits a strong tendency for genetic recombination, particularly in the intergenic segment between ORF1 and ORF2 [23]. Molecular clock analyses estimate PCV2’s evolutionary rate at ~3.5 × 10^−3^ substitutions/site/year, driven primarily by mutation pressure from continuous transmission in high‐density farming environments [24]. Epidemiological studies in Sardinia, Italy, have documented PCV2 transmission dynamics, revealing a cross‐species network involving free‐range domestic pigs and wild boars, which may accelerate genetic diversification and dissemination across ecological populations [24, 25].

PCV3 follows an evolutionary trajectory distinct from PCV2. A 2018–2022 epidemiological study in China, based on ORF2 sequence phylogeny, classified global PCV3 strains into three primary clades (PCV3a, PCV3b, and PCV3c), with Chinese isolates predominantly belonging to PCV3a (68.2%) and PCV3b (31.8%) [26]. PCV3a shows high genetic stability (nucleotide similarity: 97.8%–99.2%), while PCV3b exhibits greater diversity (96.4%–98.1%). Selection pressure analysis identified multiple positively selected sites (*ω* = 2.31) in the PCV3 Cap protein, suggesting amino acid mutations at these sites may enhance immune escape, facilitating persistent infection and viral spread [27]. Unlike PCV2, PCV3 prevalence is significantly higher in wild boar populations in China than in intensive farming systems, indicating a potential adaptation to low‐density environments, whether agricultural or wildlife [28].

**Figure 2:**
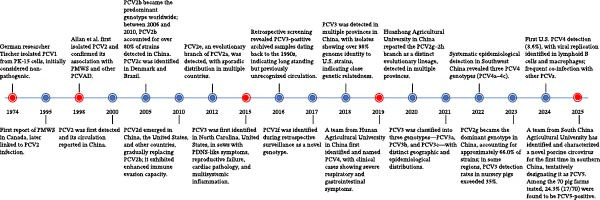
Timeline of key discoveries and epidemiological milestones of PCV1–4. Chronological summary from the first isolation of PCV1 in 1974 to recent reports of PCV4 circulation in domestic pigs and wild boars. Major milestones include the identification of PCV2 as the causative agent of PCV–associated diseases, the global genotype shifts within PCV2, the discovery of PCV3 in 2016, and the emergence of PCV4 in 2019. Red nodes indicate the first discovery of each PCV, while blue nodes denote subsequent epidemiological or evolutionary milestones. This visualization highlights the progressive recognition of PCVs and their global dissemination over the past five decades.

PCV4 exhibits unique epidemiological traits. A retrospective Spanish study analyzing 302 wild boar lymph node samples demonstrated widespread PCV4 circulation in European wild boars since at least 2011, with deeper phylogenetic analyses tracing its introduction to ~2000 [29]. PCV4 has formed two primary evolutionary branches: a China‐specific lineage and an international lineage that includes isolates from the US, Spain, and other Asian regions. Spanish isolates form a distinct monophyletic cluster with minimal genetic distances (0.00%–1.76%), indicating prolonged, relatively slow adaptive evolution in the region [30]. Additionally, PCV4 has been detected in wild boars and outdoor‐reared domestic pigs in several Asian regions but has not been reported in intensively raised pig populations to date, suggesting a possible association with outdoor or wildlife‐related environments [31]. In contrast, PCV5 has so far been represented by only a limited number of sequences, precluding robust phylogenetic or evolutionary inference [15]. For a chronological overview of PCV1‐5 discoveries and evolutionary milestones, see Figure 2.

The apparent survival advantage of PCV3 and PCV4 in low‐density or outdoor settings may be related to their ability to establish persistent infections with prolonged viremia, to cross the placenta and maintain vertical transmission cycles in small herds, and to withstand environmental inactivation in shared habitats used by domestic pigs and wildlife. In wild boars, infrequent but repeated contacts among family groups may favor viruses capable of long‐term carriage and intermittent shedding. However, experimental data on transmission efficiency and environmental stability remain limited, and the relative contributions of these mechanisms to PCV3/4 maintenance under low host density remain unclear.

### 2.2. Pathogenicity of Different PCV Types

#### 2.2.1. PCV2 Pathogenic Mechanisms

PCV2 infects multiple pig cell types, including macrophages, lymphocytes, and epithelial cells [32–34]. Viral entry occurs via receptor‐mediated endocytosis, and replication occurs in the nucleus. PCV2 infection triggers pathological changes, including lymphocyte depletion, macrophage activation, and cytokine dysregulation [32, 34, 35]. In PCV2‐SD, the virus causes severe wasting, lymphoid atrophy, and multisystemic lesions. The underlying pathogenic processes remain intricate and only partially defined, involving both direct viral cytopathic effects and indirect immunopathological responses.

Recent studies show that PCVs modulate host innate immunity by disrupting the cyclic GMP‐AMP synthase (cGAS)–stimulator of interferon genes (STING)–type I interferon (IFN‐I) axis. The replication‐associated proteins (Rep) of PCVs competitively bind cytosolic DNA and inhibit cGAS oligomerization, suppressing interferon production [36]. This strategy contributes to viral immune evasion and the persistence of infection. PCV2 also induces multiple regulated cell death pathways [32]. Markers of pyroptosis (e.g., caspase‐1 and gasdermin D) are elevated in PCV2–infected lymphoid tissues, particularly in severe cases of PCV2‐SD. Beyond innate immune modulation and cell death, viral genotype plays a critical role in disease severity [37]. However, available evidence remains inconsistent, and several studies suggest that strain‐specific differences may be more relevant than genotype‐level patterns. Moreover, autophagy and apoptosis are also activated, potentially regulating viral replication and immune clearance [38].

Our recent work has further clarified the roles of host metabolic and immune regulators in PCV2 pathogenesis [39, 40]. We found that HMG‐CoA reductase (HMGCR), a key metabolic enzyme, negatively regulates PCV2 infection: its inhibition or inactivation promotes viral replication and enhances PCV2–induced apoptosis in vitro and in vivo [39]. Conversely, host proteins such as tripartite motif‐containing protein 21 (TRIM21) enhance interferon production while inhibiting apoptosis, thereby facilitating viral persistence [40]. These findings highlight host metabolic enzymes and immune modulators as key determinants of PCV2 pathogenicity, with potential as targets for antiviral therapies.

Genotype‐specific differences further shape disease outcomes. PCV2d, now globally dominant, exhibits higher replication efficiency and stronger tissue tropism than PCV2a or PCV2b [41]. Positive selection has been detected at key ORF2 sites under vaccine‐induced pressure, although current evidence supports broad cross‐protection across PCV2 genotypes, with differences limited primarily to partial reductions rather than complete immune escape. Field studies frequently report the codetection of PCV2d with PCV3 in clinically affected pigs, characterized by high viral loads and lymphoid tissue damage [5].

Collectively, these mechanisms provide a mechanistic bridge to the clinical syndromes observed in the field. Lymphocyte depletion altered cytokine profiles, and dysregulated cell death in lymphoid tissues underlies the wasting, lymphoid atrophy, and systemic lesions typical of PCV2‐SD. Similarly, PCV2–induced immunosuppression compromises mucosal and systemic defenses, predisposing pigs to severe respiratory disease and enteric disorders in the context of secondary infections. In reproductive disorders, persistent viremia and virus‐associated placental or fetal lesions are consistent with impaired antiviral immunity and virus‐driven tissue damage. Thus, PCV2 pathogenesis emerges from the convergence of direct cytopathic effects, immune evasion, and immune‐mediated injury, which together shape the spectrum and severity of PCV–associated clinical outcomes.

#### 2.2.2. PCV3 and PCV4: New Insights Into Pathogenicity

First recognized in 2016, PCV3 has been linked to a broad spectrum of clinical disorders, including reproductive failure, myocarditis, PDNS–like manifestations, and systemic inflammation [6], and recent case‐definition criteria have been proposed to define PCV3–associated disease better [27]. While experimental infections often result in mild or no overt disease, field studies consistently link PCV3 to lymphoid depletion, vasculitis, and fetal mummification [6]. Its ability to persist in tissues and co‐occur with PCV2 may exacerbate pathological outcomes [5]. Moreover, PCV3 DNA has been detected in the brains and cerebrospinal fluid of stillborn piglets, as well as in fetal tissues and umbilical cords, suggesting both neurotropism and vertical transmission [42, 43]. PCV3 also interferes with innate immunity by targeting IFN‐I signaling pathways, potentially via mechanisms shared with other circoviruses [36].

PCV4, identified in 2019, has been found in pigs with respiratory, enteric, or reproductive symptoms, as well as in asymptomatic animals [14]. Its tissue tropism appears broader than that of PCV2/3, with viral DNA detected in the lungs, lymph nodes, spleen, kidneys, and intestines [42, 44]. While a causal link between PCV4 and clinical disease remains unclear, studies report coinfections with PCV2 or PCV3 in diseased pigs, which may contribute to symptom severity [45]. Importantly, PCV4 DNA has also been detected in brain and spinal cord tissues, particularly in aborted or stillborn fetuses, indicating potential neuroinvasion [42, 44, 45]. Recovery of an infectious PCV4 clone has experimentally verified its ability to replicate and cause infection in vivo, as evidenced by viremia, antibody responses, and cytokine upregulation in challenged piglets [44].

Furthermore, our experimental comparisons in piglets have revealed distinct pathogenic signatures for PCV2, PCV3, and PCV4 [42]. PCV2 caused classical wasting and lymphoid depletion, while PCV3 induced milder disease but broader tissue tropism (with viral detection in the heart, lungs, and brain). PCV4 produced unique pathological lesions, including cardiac fibrosis and renal changes, despite the absence of severe clinical symptoms. These findings indicate that while PCV3 and PCV4 are less virulent than PCV2, they can cause organ‐specific damage and contribute to subclinical or coinfection‐associated syndromes.

Overall, both PCV3 and PCV4 can cause systemic infections, with the potential for vertical transmission and neuroinvasion. Their ability to persist, interact with PCV2d, and cause organ‐specific damage highlights their possible synergistic roles in PCV–associated diseases. Combined with field and experimental evidence, our data underscore the need to recognize PCV3 and PCV4 not as benign agents but as emerging pathogens that require improved diagnostics, surveillance, and vaccine strategies.

### 2.3. Global Prevalence Trends of PCV

Genotype shifts, vaccination pressure, and interspecies transmission shape the global distribution of PCVs. PCV2 remains globally dominant, with genotype transitions from PCV2a to PCV2b (~2005) and later to PCV2d (~2013–2016) across China, the United States, and Europe [5, 12]. PCV2d now predominates worldwide due to its enhanced replication, genetic diversity, and partial vaccine escape [41], a shift likely reflecting immune selection under widespread vaccination. Its prevalence exceeds 70% in some regions [12]. Novel variants, including PCV2e and PCV2f, have been recently identified in both wild and domestic pig populations, especially across South America and China [45–47]. Recent studies have detected PCV2 in a broad range of non‐Suid hosts, including wild ruminants, carnivores, and rodents, indicating frequent cross‐species spillover, although most such findings likely reflect limited epidemiologically insignificant dead‐end infections [48–52].

PCV3 has become widespread, with prevalence estimates ranging from 10% to over 50% [6]. It has been reported in multiple species, including pigs, wild boars, ruminants, dogs, and arthropods, often in coinfection with PCV2 [53]. Vertical transmission and persistent infections raise concerns for long‐term herd health. PCV4 shows a relatively limited yet increasing occurrence, being identified in pigs with respiratory or reproductive disorders, as well as in wild boars [45]. Holgado‐Martín et al. found 33.7% of wild boar lymph node samples positive for PCV4 [54]. Notably, coinfection with PCV2/3 is common [45]. By contrast, PCV5 has been detected only sporadically to date, and no reliable prevalence estimates are currently available.

In summary, PCV2d is the dominant strain globally, while PCV3 and PCV4 cocirculate at varying levels, sometimes across different species. Ongoing surveillance is critical to tracking prevalence trends and adapting vaccination strategies.

## 3. Immunosuppression and Coinfection

### 3.1. Immunosuppression Induced by Porcine Circovirus

PCV2 is well recognized for its immunosuppressive effects in swine [22, 37]. It infects macrophages, lymphocytes, and dendritic cells, impairing phagocytic and signaling functions and inducing apoptosis. Infected macrophages release high levels of interleukin‐6 (IL‐6) and tumor necrosis factor‐*α* (TNF‐*α*), contributing to cytokine imbalance and immune dysfunction [55–58]. Furthermore, PCV2 hampers antigen presentation by suppressing major histocompatibility complex class II (MHC‐II) expression and reducing the levels of costimulatory molecules, such as CD80 and CD86, on antigen‐presenting cells, thereby weakening T cell activation and diminishing the immune response to vaccination [59].

Beyond general immune disruption, PCV2 modulates specific immune cell subsets and signaling pathways. Recent findings highlight its suppression of T helper 17 (Th17) responses, resulting in reduced IL‐17A production and compromised mucosal immunity [60]. Natural killer (NK) cell cytotoxicity is also diminished, particularly in lymphoid tissues, facilitating viral persistence [61]. Advances in molecular profiling further reveal that PCV2 alters host microRNA expression (e.g., miR‐139‐5p and let‐7e), which in turn regulates antigen processing and cytokine signaling [61, 62].

In addition to PCV2, PCV3 may also contribute to immunosuppression [6, 32, 34, 63]. It reduces interferon‐*β* (IFN‐*β*) production and suppresses lymphocyte proliferation in vitro, potentially aiding immune evasion and viral persistence [32, 64]. Notably, persistent PCV3 viremia has been associated with reproductive and neonatal syndromes, although the underlying mechanisms remain unclear and may involve systemic immune dysregulation rather than a direct causal effect [6, 65, 66]. Collectively, these findings demonstrate that PCVs employ diverse and evolving immunosuppressive strategies that target both innate and adaptive immunity, thereby facilitating secondary infections and reducing vaccine efficacy in affected herds.

### 3.2. Consequences of Immunosuppression in Pigs

PCVs, especially PCV2, are widely considered capable of inducing immunosuppression, predisposing pigs to severe clinical complications [6, 22, 67]. Pigs with PCV2–induced immune dysfunction are highly susceptible to secondary infections with bacteria such as *Streptococcus suis* and *Escherichia coli* [68, 69], as well as viruses including porcine parvovirus (PPV), porcine reproductive and respiratory syndrome virus (PRRSV), porcine epidemic diarrhea virus (PEDV), etc. [67]. These coinfections typically lead to exacerbated symptoms, higher pathogen loads, and increased mortality.

Recent studies suggest synergistic pathogenicity in PCV2‐PRRSV, PCV‐African swine fever virus (ASFV), PCV2–pseudorabies virus (PRV), or PCV2‐PPV coinfections, characterized by more severe lung lesions, elevated viremia, and pronounced lymphoid depletion compared to single infections [70–73]. Similar outcomes have been observed in PCV2‐*Mycoplasma hyopneumoniae* coinfection models, where respiratory pathology and growth suppression are markedly intensified [71]. Additionally, PCV2–associated immunosuppression may contribute to reduced vaccine efficacy, as infected pigs often exhibit weaker antibody responses, likely linked to defective antigen presentation and T cell priming, leading to vaccine failure even when immunization protocols are followed correctly [72].

Emerging evidence suggests that PCV3 may also diminish immune responsiveness, particularly in chronically infected herds [74]. The virus has been identified in both vaccinated and unvaccinated pigs exhibiting reproductive issues and systemic inflammation, suggesting that PCV3–associated immune modulation may persist despite vaccination. This potential for ongoing immune dysregulation helps explain why PCV coinfections can complicate vaccine responses and reduce the overall effectiveness of immunization programs. Therefore, PCV–induced immunosuppression not only worsens disease through coinfection but also poses a major barrier to effective immunization and disease control in modern pig farming.

### 3.3. Coinfections and Their Impact

Coinfections involving PCVs are increasingly recognized as important contributors to disease complexity, reflecting interactions that extend beyond simple pathogen overlap.

#### 3.3.1. Common Coinfection Combinations

PCVs frequently coinfect pigs with a range of bacterial and viral pathogens, resulting in complex, often synergistic clinical outcomes (Table 1). Among these, recent studies continue to highlight that PCV2‐PRRSV coinfection is a well‐documented pairing that exacerbates respiratory and reproductive disorders [75].

**Table 1:** Recent studies on PCV2, PCV3, and PCV4 coinfections with other pathogens (2015–2025).

Coinfection	Key synergistic mechanisms	Exacerbated clinical outcomes	References
PCV2 + PRRSV	High coinfection rates (26%–27%) have been reported in China; PRRSV enhances PCV2 replication and immunosuppression	Severe PRDC with higher viremia, pneumonia, mortality, and poor vaccine response	[75]
PCV2 + PRV	Coinfection activates NF‐*κ*B, MAPK, and NLRP3; suppresses IFN‐*β*/JAK‐STAT signaling; and enhances PRV replication	More severe neurological and respiratory disease and high piglet mortality	[73, 76]
PCV2 + PPV1	Frequently linked to SMEDI, PPV facilitates PCV2 infection of embryos, worsening outcomes	Increased abortions, stillbirths, fetal mummification	[67, 70]
PCV2 + swine influenza virus (SIV, H1N1)	PCV2b reduces H1N1 replication in epithelial cells but enhances it in macrophages, altering the IFN response	Compounded respiratory illness; worsened pneumonia in coinfected pigs	[67]
PCV2 + *M. hyopneumoniae*	PCV2 immunosuppression promotes wider *M. hyo* colonization; dual infection worsens lesions	Severe chronic pneumonia, poor growth	[71]
PCV2 + *M. hyorhinis*	Sequential infection increases PCV2 load and cytokine levels (TNF‐*α* and IL‐6) and exacerbates lung inflammation	Polyserositis, pneumonia, fever, and growth retardation	[77]
PCV2 + *G. parasuis*	Coinfection exacerbates Glässer’s disease, leading to more severe inflammation and an impaired antibody response	Severe polyserositis, pneumonia, and higher mortality	[78]
PCV2 + *P. multocida*	Outbreak study: PCV2a and virulent *P. multocida* type D isolated together	Severe bronchopneumonia, high morbidity, and mortality	[79]
PCV2 + ASFV	First codetection in ASFV–positive pigs (Mongolia and Indonesia); multiple PCV2 genotypes present	May worsen ASF outbreaks and complicate diagnosis and control	[70]
PCV2 + PCV3	Coinfection rates are 16% in the US; suspected synergy but no recombination	Overlapping PCV–associated diseases like syndromes and intensified lesions	[80]
PCV3 + PRRSV	Widely codetected in PRRS outbreaks, PCV3 may aggravate PRRS lesions	More severe respiratory disease, reproductive losses, and poor vaccine response	[80]
PCV3 + PPV7	High coinfection rates in sows with reproductive failure	Increased abortions, stillbirths, and infertility	[74]
PCV3 + *M. suis*	Vietnam study: ~30% of PCV3–positive sows also tested positive for *M. suis*	Reproductive failure with anemia and neonatal weakness	[81]
PCV3 + ASFV	First detection of ASFV–infected pigs in Mozambique	May complicate ASF epidemiology and outcomes	[70]
PCV4 + PCV2/3	Most PCV4–positive pigs are coinfected; no cross‐reactivity with PCV2/3 antibodies	PCV2‐SD–like wasting, PDNS, reproductive disorders; role still unclear	[82]

Coinfection with PPV is strongly linked to reproductive failure, manifested as fetal losses such as resorption and mummification [70, 74]. In respiratory diseases, simultaneous infection with PCV2 and *Mycoplasma hyopneumoniae* worsens lung lesions and growth retardation, as both pathogens impair the host’s respiratory defense mechanisms [71].

Recent observations have further reported PCV3 coinfection with PRRSV, PCV2, or PEDV, especially in cases of reproductive and neonatal diseases [80], suggesting emerging cocirculation patterns with potential immunomodulatory relevance.

#### 3.3.2. Synergistic Effects on Disease Severity

Coinfections involving PCVs often exhibit synergistic interactions that amplify disease severity by enhancing viral replication, suppressing the immune response, and cumulatively causing tissue damage [70]. In PCV2‐PRRSV dual infections, for example, PRRSV impairs pulmonary macrophage function, while PCV2 concurrently inhibits adaptive immune responses, together driving more severe pneumonia and prolonged viremia [83].

A comparable synergistic pattern is observed in PCV2‐PPV infections, where virus‐induced apoptosis in lymphoid tissues facilitates viral dissemination to the uterus and placenta, thereby exacerbating reproductive losses [70, 84–86]. In triple infections with PCV2, PRRSV, and *Mycoplasma hyopneumoniae*, pigs develop multisystemic inflammation and poor growth performance even after vaccination [71], highlighting the difficulty of managing multipathogen disease in field settings.

Our studies have further revealed that PCV2 and PRV coinfection aggravates disease progression by triggering endoplasmic reticulum stress (ERS) and initiating the unfolded protein response (UPR) through activation of the PERK–eIF2*α*–ATF4–CHOP and IRE1–XBP1–EDEM signaling cascades, which collectively promote apoptosis and tissue damage [76]. Additionally, we found that PCV2‐PRV coinfection amplifies immunosuppressive and inflammatory responses through the NF‐*κ*B, JAK/STAT, MAPK, and NLRP3 pathways, contributing to more severe neurological and respiratory pathology, as well as increased piglet mortality in vivo [73].

While the precise mechanisms underlying PCV3‐PCV4 synergy remain unclear, recent field observations suggest their cocirculation with PCV2 or PRRSV may intensify clinical manifestations, delay recovery, or result in atypical disease phenotypes [6, 63, 80, 87].

Together, these findings illustrate that PCV–related coinfections are not merely additive to the need for integrated diagnostic and control strategies.

## 4. Cap Protein Immunogenicity

### 4.1. Structure and Function of the Cap Protein

Encoded by ORF2, the Cap protein is the sole structural component of PCVs, forming the virus’s icosahedral capsid (Table 2) [88]. It plays essential roles in genome packaging, nuclear localization, and host receptor interaction. Structurally, the Cap protein includes a positively charged N‐terminal nuclear localization signal (NLS), one or more DNA–binding domains that interact with the viral genome, and several surface‐exposed loops that constitute the major conformational antigenic sites. In PCV2, for example, loop regions comprising key amino acid positions within ORF2 form dominant neutralizing epitopes, whereas in PCV3 and PCV4, the overall capsid architecture is conserved, but specific substitutions in these loops and in the NLS motif have been reported, potentially altering receptor usage, nuclear trafficking efficiency, and epitope exposure among species and genotypes [89, 90].

**Table 2:** Structural and immunological comparison of PCV2, PCV3, and PCV4 Cap proteins (2015–2025).

Feature	PCV2	PCV3	PCV4
Cap gene length and domains	233–236 aa; N‐terminal arginine‐rich NLS; heparan sulfate‐binding motif present	214 aa; retains N‐terminal basic NLS; lacks XBBXBX heparan sulfate‐binding motif	228 aa; NLS at residues 4–37; ORF2 length distinct from PCV2/3
Epitope conservatism and variation	Multiple immunodominant Cap regions (e.g., aa51–84, 113–139, 161–207, and 228–233); intraspecies conserved; distinct from PCV3/4	Several linear B cell epitopes identified (cores at aa57–61, 140–146, and 161–166); minimal overlap with PCV2 epitopes	Predicted surface‐loop epitopes (5B cell sites); unique antigenic sites (no cross‐reactivity with PCV2/3)
VLP assembly and expression	Cap self‐assembles into ~17–20 nm VLP (*T* = 1 icosahedral, 60 subunits); expressed in baculovirus or *E. coli* for vaccines	Recombinant Cap forms VLP (~10 nm observed); efficiently expressed in *E. coli* (codon‐optimized)	Cap efficiently self‐assembles into VLP (~20 nm) in *E. coli*; VLPs enable serological test development
Neutralizing antigenicity and cross‐reactivity	High neutralizing antibody titers induced by infection/vaccines; cross‐protects all PCV2 genotypes; no cross‐neutralization with PCV3/PCV4	Distinct antigenicity; PCV2–derived immunity offers no protection; PCV3 infection elicits homologous neutralizing antibodies (no cross‐protection with PCV2)	Antigenically divergent; PCV2/PCV3 antisera show negligible reactivity to PCV4 Cap; presumed lack of cross‐neutralization
Key residues/3D structure	Cryo‐EM solved Cap structure (*T* = 1 symmetry); surface loops (e.g., FG‐loop aa168–180) mutate frequently (antigenic drift)	Homology model: different surface loop conformation (exposed NLS region) vs. PCV2; Cap mutations A24V/R27K define PCV3a‐c clades	3D structure (modeled) shows distinct surface loops (BC, CD, DE, EF, and GH) vs. PCV2; surface enriched in polar residues; single residue 27 (S/N) distinguishes PCV4a vs. PCV4b
Host sensing and inflammatory pathways	CpG–rich DNA activates TLR9 and cGAS–STING (type I IFN induction); counters via IL‐10 induction (p38 MAPK/NF‐*κ*B) and IRF3 inhibition (net immunosuppressive effect)	Innate sensing differs (CpG motif disparity vs. PCV2); PCV3 Cap inhibits type I IFN (binds G3BP1 and KPNA1/STAT2); typically milder inflammation	Sensed by DNA sensors (TLR9 and cGAS–STING) similar to PCV2/3; PCV4 Rep disrupts cGAS; coinfections exacerbate inflammation (pathways under study)
Immune responses	Infection elevates IL‐8, IL‐1*β*, and IL‐10 in pigs, leading to lymphoid depletion and immune cell apoptosis (PCV–associated disease pathology). Vaccines elicit strong neutralizing antibodies and IFN‐*γ* (Th1) responses	Can induce PDNS–like lesions and chronic inflammation (e.g., lymphoplasmacytic myocarditis); generally milder clinical disease than PCV2; high seroprevalence (frequent subclinical infections)	Experimental PCV4 infection causes multiorgan lesions (e.g., cardiac fibrosis and nephropathy); triggers a distinct cytokine profile vs. PCV2/3; specific antibody responses detected in infected pigs (serological evidence of exposure)

Advances in recombinant protein expression and cryo‐electron microscopy (cryo‐EM) have enabled detailed characterization of Cap across PCV genotypes. Bi et al. [88] characterized the recombinant Cap protein of PCV3, demonstrating its ability to form VLPs. These PCV3 VLPs induced strong humoral and cellular immune responses [88, 90, 91], indicating that conserved structural motifs are critical for both capsid formation and immunogenicity. Comparative analyses across genotypes have further revealed amino acid substitutions in surface loops, particularly in PCV2d, that may alter epitope exposure and immune recognition. This antigenic drift helps explain variations in virulence and vaccine responsiveness among genotypes. Liu et al. [92] developed a blocking ELISA targeting genotype‐specific Cap epitopes, confirming the functional relevance of these mutations. Comparative analyses of PCV2a and PCV2d Cap have identified genotype‐specific substitutions in surface loops that modify local charge and hydrophobicity, whereas PCV3 and PCV4 Cap proteins retain the overall *T* = 1 icosahedral symmetry but show lower sequence identity and distinct loop conformations. These interspecies and intergenotype differences in Cap topology are likely to contribute to the observed variability in antigenicity, neutralization profiles, and cross‐protection among PCV strains.

These findings highlight that the structural domains of the Cap protein are not only critical for capsid integrity but also shape immunogenicity and immune escape. Such insights clarify mechanisms of immune modulation and guide the rational design of genotype‐specific diagnostics and next‐generation vaccines.

### 4.2. Immunogenicity of Cap Protein

#### 4.2.1. Induction of Immune Response

The Cap protein is the primary immunogen driving host immune recognition during PCV infection [91]. It stimulates both humoral and cellular branches of the immune system, establishing itself as a core element in diagnostic applications and vaccine development.

For PCV2, extensive research has demonstrated that vaccines based on Cap‐derived VLPs effectively provoke strong neutralizing antibody production and activate both CD4^+^ and CD8^+^ T lymphocytes [93, 94]. Experimental vaccination with recombinant PCV2 Cap‐VLPs has been shown to induce high levels of Cap‐specific serum IgG and a balanced Th1/Th2 cytokine response, reflecting potent systemic and mucosal immune protection [95].

For PCV3, recent studies have confirmed the immunogenicity of its Cap protein [96]. Recombinant PCV3 Cap formed well‐structured VLPs and promoted lymphoproliferation, IFN‐*γ*, and IL‐10 secretion, suggesting coordinated activation of both innate and adaptive immunity [96].

In contrast, emerging evidence suggests that the PCV4 Cap protein is less immunogenic [16]. Researchers have expressed recombinant PCV4 Cap in insect cells or *E. coli* [16, 97], demonstrating that it triggers a measurable IgG response but only weakly stimulates T cell–associated cytokines, indicating a modest immunostimulatory profile relative to PCV2 and PCV3 [97]. Our team further found that antibodies generated against recombinant Cap proteins of PCV2 to PCV4 can cross‐react with different PCVs [90]. Together, these findings suggest that the Cap proteins of PCVs exhibit conserved immunogenicity, with PCV4 potentially showing reduced potency. Such comparative insights are crucial for optimizing vaccine design and evaluating the cross‐protective potential among these viruses.

#### 4.2.2. Factors Affecting Immunogenicity

Multiple factors influence the immunogenicity of the PCV Cap, including amino acid variation, posttranslational modifications, and the host’s immune status [88].

At the genetic level, PCV genotype diversity plays a pivotal role. Mutations in the surface‐exposed loops of Cap, especially in PCV2d and PCV2e, can alter key antigenic epitopes, potentially reducing vaccine efficacy and diagnostic sensitivity [1]. Liu et al. [92] developed a blocking ELISA targeting genotype‐specific epitopes, demonstrating that these substitutions contribute to immune escape and diagnostic underperformance.

Posttranslational modifications, particularly N‐linked glycosylation, can modulate the immunogenicity of PCV2 Cap. Gu et al. [98] investigated the impact of a putative glycosylation site (N143YS) in the Cap protein by comparing wild‐type and glycosylation‐deficient constructs in a mouse DNA vaccination model. Their results showed that deleting this glycosylation site significantly enhanced Cap‐specific T cell activity, increased CD8^+^ T cell percentages, elevated IFN‐*γ* levels, and improved IgG2a/IgG1 antibody profiles, indicating stronger cellular and humoral immunity. These findings suggest that N‐glycosylation may mask critical antigenic sites or alter epitope exposure and that glycosylation status should be carefully considered in the design of recombinant Cap‐based vaccines. Furthermore, our work revealed that the NLS, particularly its motifs, affects VLP formation and immunogenicity [89].

In vivo, the host’s immune status profoundly influences vaccine responsiveness. Factors such as age, maternal antibody interference, and immunosuppression from coinfections (e.g., PRRSV or *Mycoplasma hyopneumoniae*) can blunt both humoral and cellular immune responses to Cap‐based vaccines [5, 41]. According to a systematic review by Nautrup et al., elevated levels of maternally derived antibodies (≥8 log_2_ IPMA titer) can partially impede vaccine‐induced immune activation, resulting in delayed seroconversion and slower antibody maturation; nevertheless, vaccination administered at 3 weeks of age remained effective in reducing viremia and enhancing growth outcomes [99].

Together, these insights underscore the importance of genotype‐specific surveillance, structural epitope mapping, and host condition assessment to guide rational vaccine design and epitope engineering, thereby achieving broader protective coverage.

### 4.3. Applications of Cap Protein Immunogenicity Research

The strong immunogenicity of the Cap protein makes it a core component of both diagnostic assays and vaccine development for PCVs [90, 92]. Cap‐based diagnostic methods, particularly ELISA platforms, are widely used for serological surveillance. For instance, Park et al. [100] established an in‐house ELISA using rabbit‐derived polyclonal antibodies specific for PCV2d, achieving excellent sensitivity and specificity and successfully validating its application in both guinea pig and pig serum samples.

The Cap protein also serves as the primary immunogen in subunit and VLP vaccines [88, 89]. Several studies have shown that oral delivery platforms based on recombinant *Bacillus subtilis*, *Lactobacillus casei*, or attenuated *Salmonella* expressing PCV2 Cap or Cap‐VLPs can effectively induce both systemic and mucosal immune responses, offering promising noninjection strategies against PCV2d [94]. Recent studies have further demonstrated that VLPs derived from PCV3 and PCV4 exhibit strong immunogenic potential, capable of inducing robust antibody responses and mucosal immunity in vivo [90, 91, 101]. Our team also found that both monovalent and trivalent vaccines formulated with recombinant Cap proteins induced enough immune responses against diverse PCV strains [90, 102]. These findings highlight their promise as subunit vaccine candidates for emerging PCV genotypes.

Given the increasing genetic diversity of PCV genotypes, there is growing interest in vaccine approaches that broaden antigenic coverage to address strain variability. Accordingly, novel Cap‐based platforms are being designed to improve cross‐protection [90, 93, 103]. One example involves the construction of a bivalent nanoparticle vaccine combining PCV2 Cap and PRRSV–neutralizing epitopes, which successfully elicited dual antigen‐specific immune responses in mice and piglets [104]. This type of multivalent vaccine addresses the need for broad‐spectrum protection in the coinfection scenarios commonly encountered in field conditions. Li et al. [105] engineered a recombinant PRV that simultaneously expresses PCV2 and PCV3 capsid proteins. This recombinant construct elicited high titers of virus‐specific antibodies and cytokines while markedly reducing viral load and tissue damage in piglets, demonstrating a promising platform for the development of multivalent PCV vaccines.

Collectively, these findings emphasize that Cap protein immunogenicity research not only enhances diagnostic and prophylactic strategies but also guides the rational design of multivalent and genotype‐adapted vaccines to control emerging PCV strains.

## 5. Vaccine

### 5.1. Traditional Vaccination Strategies for Porcine Circovirus

Early PCV control efforts primarily relied on inactivated and live‐attenuated vaccines, especially targeting PCV2 (Table 3) [125, 126]. Inactivated vaccines, produced via chemical or physical inactivation and formulated with adjuvants, offered a favorable safety profile but typically required multiple doses for optimal protection, particularly under field conditions. Live‐attenuated vaccines could stimulate stronger immunity with fewer doses, but they raise concerns about reversion to virulence and recombination with circulating strains. While some live vaccines demonstrated efficacy, studies have highlighted the genetic risks associated with their use, necessitating close surveillance.

**Table 3:** Vaccines against porcine circovirus have been developed in the past decade (2015–2025).

Vaccine	Clinical protection	Immune mechanism	References
Traditional vaccines			
Live‐attenuated chimeric PCV1‐2b (PCV1 backbone expressing PCV2b Cap)	Protects against PCV2b/PCV2d; reduced viremia and shedding; decreased tissue viral load; more efficacious than commercial comparator	High PCV2–neutralizing antibody; strong IFN‐*γ* T cell response (Th1); minimal lesions	[106]
Live chimeric PCV1‐2a carrying PRRSV epitopes	Dual protection (PCV2 + PRRSV epitopes); neutralization against both pathogens	Bivalent response: PRRSV–neutralizing antibodies and anti‐PCV2 antibodies; IFN‐*γ* T cell activation	[107]
Inactivated chimeric PCV1‐2 (PCV2b/PCV2d, whole virus)	Similar efficacy of 2b‐ and 2d‐based vaccines; prevented disease; reduced PCV2b viremia	High PCV2 antibody titers; blocked systemic spread; strong, comparable humoral responses	[108]
Bivalent killed vaccine: PCV2d + *Mycoplasma hyopneumoniae*	Improved growth performance; decreased lung and lymphoid lesions; reduced PCV2d viremia and *M. hyo* load in coinfected pigs	High PCV2d–neutralizing antibody; increased PCV2d–specific IFN‐*γ*‐secreting cells; increased *M. hyo*–specific IFN‐*γ*	[109]
Subunit and VLP vaccines			
PCV2d VLP (baculovirus/Sf9)	Complete protection against PCV2d; cross‐neutralizes PCV2b; reduced viremia and lesions	High IgG and neutralizing antibodies; strong IFN‐*γ* (Th1); improved weight gain	[110]
Intradermal PCV2d VLP (needle‐free)	Immunogenicity superior to IM; 1.3‐fold higher neutralizing titers; dose‐sparing	Intradermal targeting of dendritic cells; strong IgG and neutralization with optimized IMS adjuvant	[111]
PCV2d subunit (*E. coli* platform)	Demonstrated industrial‐scale feasibility; retained antigenicity	High‐yield Cap production; one‐step purification; recombinant Cap immunogenic	[112]
PCV2 VLP (*Kluyveromyces* yeast)	Mice: reduced challenge titers in liver/spleen; protective	Very high‐yield VLP; strong anti‐PCV2 IgG	[113]
Plant‐based PCV2a VLP (*Nicotiana benthamiana*)	Protection across PCV2a/2b/2d/2e, even with PRRSV cochallenge; reduced viremia and lesions	High genotype‐specific neutralizing antibodies; increased IFN‐*γ*–secreting cells (Th1)	[114]
PCV2 multiepitope peptide nanovaccine	Mice: uniformly high antibodies; single‐dose efficacy with polymer nanoadjuvant	Strong B cell response (Th2–biased) with T helper epitope support	[115]
PCV3 Cap VLP with PCV2 neutralizing epitope (chimeric)	Mice: reduced PCV3 lung viral load and lesions; sera neutralized PCV3 and PCV2	High PCV3‐Cap antibodies; cross‐neutralization; robust IFN‐*γ*	[96]
Fusion Capsid subunit (PCV2+PCV3+PCV4)	Mice: high IgG to all three; neutralized PCV2/PCV3; protected against lung pathology	Broad B cell repertoire; strong IFN‐*γ* and T cell activation	[97]
PRV–vectored PCV vaccines (pseudorabies vectors: PCV2 Cap; PCV3 Cap; PCV2 + PCV3; ± IL‐4)	Piglets/mice: reduced PRV/PCV loads; 100% survival vs. PRV; PCV2d vector outperformed commercial vaccine	High PCV–neutralizing antibodies; mixed Th1/Th2 (elevated IFN‐*γ*/IL‐2/IL‐4); IL‐4 version further enhanced antibody responses	[105]
NDV–vectored PCV2 vaccine (recombinant Newcastle disease virus expressing Cap)	Mice fully protected; pigs: long‐lasting neutralizing antibodies; no viremia after PCV2d challenge	High neutralizing antibodies (2a/2b/2d); elevated CD4^+^/CD8^+^ ratios; increased IFN‐*γ* and TNF‐*α*; decreased IL‐10	[116]
Cap‐E2 Spy‐VLP (PCV2 VLP displaying CSFV E2)	Mice: higher E2‐IgG than soluble E2; PCV2 VLP immunogenicity preserved	Multivalent antigen display; enhanced DC uptake; strong antibody induction	[117]
*Lactococcus lactis* GEM‐PA‐Cap (PCV2)	Piglets: higher PCV2 antibody; greater weight gain; milder fever/lesions after challenge	Surface display induced mucosal and systemic immunity; neutralizing antibodies	[118]
*Bacillus subtilis* spore‐display Cap (PCV2)	Mice: high intestinal IgA and serum IgG; robust mucosal/systemic immunity	Gut‐associated lymphoid tissue activation; elevated IL‐1*β*, IL‐6, and IFN‐*γ*; T cell proliferation	[119]
DNA and RNA vaccines			
rAAV8‐Cap (PCV2)	Mice: strong, durable humoral, and cellular immunity	Sustained in vivo Cap expression; Th1–biased (IFN‐*γ*); neutralizing antibodies	[120]
PCV2d ORF2 DNA + C3d‐P28 fusion	Cross‐protective vs. PCV2b and PCV2d; reduced viremia and lesions	Potent neutralizing antibodies; increased PCV2–specific IFN‐*γ* cells (Th1)	[121]
Optimized PCV2 DNA (mutant Cap + chemokines; PEI nanoliposome)	Mice: reduced lung viral load and lesions; improved survival vs. wild‐type Cap DNA	Higher antibody; balanced Th1/Th2; increased IFN‐*γ*; decreased IL‐10; chemokines enhanced APC recruitment	[122]
Bivalent PCV2a/2b DNA (fusion or codelivery)	Mice/pigs: antibody to both genotypes; reduced heterologous viremia	Broadened neutralizing repertoire; increased IFN‐*γ* T cells responsive to both genotypes	[95]
Alphavirus replicon plasmid (CSFV E2‐Erns + PCV2 Cap‐Rep)	Mice: high neutralizing antibodies to CSFV and PCV2	High antigen expression; strong B‐ and T cell activation	[123]
RNA particle (alphavirus‐like replicon) for PCV	Concept stage; reports of reduced PCV2 viremia in prototypes	High intracellular antigen expression; strong CD8^+^ T cell and antibody responses; rapid adaptability	[124]

Due to these concerns, industry trends have shifted toward safer platforms such as subunit and VLP–based vaccines. Nevertheless, traditional inactivated and live vaccines continue to serve as valuable tools in experimental studies and regions with limited access to next‐generation vaccines.

### 5.2. Novel Vaccine Developments in the Past Decade

#### 5.2.1. Subunit and VLP Vaccines

Subunit vaccines based on recombinant Cap proteins and VLP vaccines represent the leading next‐generation immunization strategies [91, 101]. Both approaches are valued for their safety, immunogenicity, and adaptability for multivalent formulations.

Subunit vaccines consist of purified or recombinant Cap proteins formulated with adjuvants. They are safe (as no live virus is involved) yet can elicit strong humoral and cellular responses. For PCV2, a plant‐derived Cap vaccine provided cross‐protection against PCV2d and improved pig growth in field trials [127]. Fusion of PCV2 Cap with CD154 enhanced both antibody and T cell responses [128]. Nanoparticle carriers, including CpG–loaded VLPs, metal–organic frameworks, and Zera particles, consistently boosted neutralizing antibody titers and T cell immunity [93, 129–131]. For PCV3, Cap subunit vaccines produced in gene‐edited insect cells showed higher protein yield and immunogenicity [132]. In parallel, *E. coli*–expressed PCV3 Cap successfully assembled into VLP–like structures that induced strong humoral and cellular responses in mice [101]. These examples highlight the versatility of subunit strategies across different expression systems.

VLP vaccines are based on Cap’s ability to self‐assemble into VLPs that mimic native virion structure without carrying viral genomes. This ensures excellent safety while preserving conformational epitopes critical for immune recognition. For PCV2, Wu et al. produced immunogenic VLPs in *E. coli*, while Duan et al. [113] achieved higher yield and cost efficiency using *Kluyveromyces marxianus* compared to baculoviral‐based systems. For PCV3, Chang et al. [133] demonstrated correct VLP assembly in a baculoviral system, confirmed by monoclonal antibody recognition. For PCV4, we found that intact NLSs were essential for VLP assembly, with N‐terminal modifications directly impacting both structural integrity and immunogenicity [89].

Multivalent and engineered strategies have expanded the potential of these platforms. Trivalent vaccines combining PCV2, PCV3, and PCV4 Cap proteins induced cross‐reactive immunity in mice, while formulations with aluminum or GM‐CSF‐CpG adjuvants enhanced immunogenicity [93, 97]. Beyond antigen combinations, structural engineering approaches have been introduced. Qi et al. [134] fused C3d (a complement receptor ligand) to PCV2d Cap to improve immune activation. Li et al. [135] attached elastin‐like polypeptide (ELP) tags to simplify purification and increase efficacy, and modular SpyTag/SpyCatcher systems enabled flexible VLP platforms that codeliver PCV antigens with heterologous swine viral proteins (e.g., classical swine fever virus (CSFV) E2), supporting dual or multivalent protection [117].

Together, these findings confirm that subunit and VLP–based vaccines form the backbone of next‐generation PCV immunization strategies. Their adaptability across expression systems, compatibility with engineered designs, and proven efficacy in multivalent formulations make them promising tools for addressing genotype diversity and coinfections in the field.

#### 5.2.2. DNA and RNA Vaccines

DNA and RNA vaccines are emerging platforms with the potential to revolutionize PCV vaccination [22, 95, 136]. DNA vaccines consist of plasmid DNA encoding the Cap protein; when injected into host cells, the protein is expressed, stimulating an immune response. RNA vaccines, by contrast, use mRNA encoding the Cap protein, which is delivered into host cells, where it is translated into the protein, triggering immunity. While PCV–targeting DNA and RNA vaccines are still in the experimental stage, they offer key advantages, including rapid development, high safety, and the ability to induce both humoral and cellular immune responses.

Several DNA vaccine strategies have shown promise. Guo et al. [137] demonstrated that coexpression of porcine IL‐6 with the Cap protein significantly enhanced CD4^+^ and CD8^+^ T cell responses in mice. Li et al. further optimized this approach by incorporating CpG motifs into DNA vaccines, thereby elevating neutralizing antibody levels and reducing pathological lesions in pigs [93, 138]. Similarly, Park et al. [136] evaluated Cap‐expressing DNA vaccines formulated with different adjuvants, finding that liposome‐based formulations induced superior neutralizing activity and IFN‐*γ* secretion. Meas et al. [95] also developed a bivalent DNA vaccine expressing fusion Cap proteins from PCV2a and PCV2b, which induced strong Th1‐skewed immunity and broad epitope recognition.

On the RNA vaccine front, Du et al. [123] constructed an alphavirus replicon‐based RNA plasmid encoding PCV2 Cap and Rep proteins, as well as CSFV antigens. This multivalent RNA vaccine elicited neutralizing antibodies and robust cellular responses in mice, demonstrating the feasibility of RNA–based immunization strategies for PCV.

These studies highlight the growing potential of nucleic acid vaccines in future PCV prevention, particularly for addressing genotype evolution and coinfections through flexible multivalent design.

### 5.3. Vaccine Efficacy and Field Application Challenges

Although significant advances have been made in the development of PCV vaccines, several obstacles remain regarding their protective efficacy and practical deployment. One of the major issues lies in the high genetic variability of PCVs, as the continual emergence of novel genotypes and variants can undermine vaccine‐induced cross‐protection. For example, Liu et al. [92] demonstrated that amino acid mutations within the surface‐exposed loops of the PCV2d Cap protein decrease antibody recognition generated by PCV2a–based vaccines, resulting in partial immune evasion and reduced diagnostic accuracy. Additionally, immunosuppression in pigs, whether caused by PCV2 infection or other factors, can compromise vaccine effectiveness.

Vaccine formulation, including the selection of adjuvants and antigen dosage, also plays a crucial role in determining efficacy [93]. Recent studies emphasize that current adjuvant systems may not elicit optimal immune responses under field conditions. For example, oil‐based adjuvants administered intramuscularly are often associated with variable efficacy and side effects [139]. In contrast, newer formulations, such as cationic liposome‐embedded squalene or optimized intradermal adjuvants like IMS1313, have shown superior antibody titers and reduced viremia in controlled settings [111, 140]. However, these novel systems remain underutilized due to higher costs, limited accessibility, or insufficient validation in commercial herds, hindering their widespread adoption.

In field settings, proper storage, handling, and administration are essential for ensuring vaccine performance [141, 142]. Field failures are frequently attributed to cold chain disruptions or improper dosing schedules. Maternally derived antibodies also interfere with vaccine seroconversion, especially when piglets are vaccinated too early [99, 143].

While PCV vaccines have significantly reduced disease burden, their field efficacy remains influenced by multiple factors. Beyond genetic diversity and immunosuppression, vaccine performance is shaped by adjuvant choice, antigen stability, and administration practices. Addressing these gaps requires continuous surveillance, genotype‐matched vaccine design, and improved immunization logistics.

## 6. Future Perspectives and Challenges

### 6.1. Unresolved Questions in Porcine Circovirus Research

Numerous critical questions in PCV research remain unanswered. The precise mechanisms underlying PCV2–induced immunosuppression and the roles of distinct immune cell subsets in the pathogenesis of PCV–associated diseases remain to be fully elucidated. The relationships between PCV3, PCV4, and other PCV types, along with their individual and combined impacts on pig health, also demand an in‐depth study [45]. In addition, the recent identification of PCV5 further underscores the likelihood that additional, previously unrecognized circoviruses may be circulating in pig populations, highlighting significant gaps in our understanding of PCV diversity, host range, and disease relevance. Developing more effective diagnostic tools capable of accurately detecting diverse PCV genotypes and variants is another priority. Additionally, understanding the drivers of new PCV genotype emergence and spread is crucial for predicting and preventing future outbreaks [22].

Despite decades of research, key knowledge gaps persist. A major challenge is the lack of integrative models that link molecular mechanisms with clinical disease expression under field conditions. Notably, the molecular basis of PCV–induced immunosuppression remains incompletely elucidated. Studies suggest PCVs, especially PCV2, disrupt the antigen‐presenting functions of dendritic cells and macrophages, downregulate MHC‐II expression, and alter cytokine signaling, all of which contribute to immune evasion and increased susceptibility to coinfections [59]. The roles of regulatory T cells and inflammatory mediators in disease progression also require further exploration.

The expanding genetic diversity of PCV2, particularly the emergence of PCV2d and PCV2e, poses additional challenges [46]. These variants may carry immune escape mutations that compromise immunity induced by existing vaccines and reduce diagnostic sensitivity. Recombination events and cross‐species transmission dynamics further complicate control strategies.

Finally, advancing diagnostic technologies, such as genotype‐specific qPCR, digital PCR, and epitope‐based ELISAs, is essential for precise surveillance and early detection of novel variants [45, 100]. Enhanced molecular epidemiology and experimental models are also needed to uncover the drivers of PCV evolution, persistence, and outbreaks.

### 6.2. Emerging Technologies for Research and Control

Cutting‐edge biotechnologies are transforming PCV research and disease control. NGS has become a key method for full‐genome surveillance of PCV strains, enabling rapid detection of new variants and recombination events [144]. Such approaches have also facilitated the discovery of highly divergent circoviruses, exemplified by the recent identification of PCV5 through metagenomic sequencing. These capabilities support the timely identification of mutation hotspots and emerging lineages, particularly in PCV2 and PCV3, which exhibit increasing genetic diversity.

CRISPR‐Cas9 genome editing has also proven to be a valuable tool for dissecting host‐virus interactions in PCV infection models. For example, p53 knockout in PK‐15 cells suppressed PCV2–induced S‐phase arrest and reduced viral replication [145]. Direct editing of the PCV2 genome with specific single‐guide RNAs (sgRNAs) significantly inhibited viral proliferation [146]. Similarly, CRISPR–mediated knockout of host factors such as ZC3H11A and SYNGR2 reduced PCV2 replication and identified potential resistance alleles [147, 148]. These studies provide mechanistic insights and support the future development of genetically engineered pigs with enhanced PCV resistance, though field applications remain conceptual.

Nanotechnology offers promising avenues for vaccine and diagnostic development. However, translating these experimental platforms into scalable and cost‐effective field applications remains a major barrier. PLGA nanoparticles conjugated with the PCV2 Cap enhance antigen uptake and trigger stronger antibody responses than soluble protein forms [149]. Cap‐Cat VLPs, utilizing SpyTag/SpyCatcher technology, enable multivalent antigen display and induce a balanced Th1/Th2 immunity [150]. Additionally, nanoliposomes carrying mutant Cap DNA vaccines improve immunogenicity and protection in mice [122]. These strategies support the development of more effective and adaptable PCV vaccine platforms.

Finally, big data analytics and artificial intelligence (AI) are increasingly used in epidemiological modeling of PCV outbreaks [151]. These tools help identify transmission trends and risk factors and predict regional spread, enabling more proactive disease management.

Collectively, these technologies, including NGS, CRISPR, nanotechnology, and AI, are reshaping PCV research, providing precision tools for surveillance, mechanistic discovery, and next‐generation control measures.

### 6.3. The Need for Multidisciplinary Approaches

Addressing PCV challenges requires a comprehensive, multidisciplinary strategy. This need has become particularly urgent as rapid genotype turnover and the emergence of PCV3 and PCV4 increasingly require coordinated responses across laboratory and field settings. Collaboration among veterinarians, virologists, immunologists, molecular biologists, and epidemiologists is crucial to comprehending and effectively combating these viruses. Veterinarians provide frontline insights into clinical manifestations, disease progression, and the practical limitations of current vaccines under field conditions. Virologists contribute to unraveling the molecular biology, evolution, and pathogenic mechanisms of viruses. Immunologists focus on host‐virus immune interactions and guide the development of more effective immunoprophylactic strategies. Molecular biologists play a key role in advancing diagnostic technologies, optimizing antigen design, and refining vaccine delivery platforms. Epidemiologists, through data modeling and surveillance, track emerging genotypes and guide region‐specific intervention strategies. Increasingly, integration of bioinformatics, systems biology, and precision livestock farming technologies enables more timely and targeted responses.

By fostering closer integration of these disciplines, PCV research and control efforts can become more proactive and adaptive, supporting the development of next‐generation vaccines, improved diagnostics, and sustainable disease management frameworks. This convergence of expertise is critical for anticipating future outbreaks, responding to genotype shifts, and safeguarding global swine health and production.

## Author Contributions

Conceptualization: Linzhu Ren. Writing – original draft preparation: Jiawei Zheng and Guoqing Zhang. Figure: Jiawei Zheng and Peiheng Li. Writing – review and revision: Guoqing Zhang and Linzhu Ren. Supervision: Linzhu Ren. Funding acquisition: Linzhu Ren.

## Funding

This work was financially supported by the National Key Research and Development Program of China (Grant 2022YFD1800905) and the Jilin Province Science and Technology Development Projects (Grant 20230508088RC).

## Disclosure

All authors have read and agreed to the published version of the manuscript. The funders played no part in study design, data collection and analysis, publication decision‐making, or manuscript preparation.

## Ethics Statement

The authors have nothing to report.

## Consent

The authors have nothing to report.

## Conflicts of Interest

The authors declare no conflicts of interest.

## Data Availability

All data generated or analyzed during this study are included in this published article.
